# Orexin 2 receptor (OX2R) protein distribution measured by autoradiography using radiolabeled OX2R-selective antagonist EMPA in rodent brain and peripheral tissues

**DOI:** 10.1038/s41598-022-12601-x

**Published:** 2022-05-19

**Authors:** Kayo Mitsukawa, Haruhide Kimura

**Affiliations:** Neuroscience Drug Discovery Unit, Research, Takeda Pharmaceutical Company Limited, 26-1, Muraoka-Higashi 2-Chome, Fujisawa, Kanagawa 251-8555 Japan

**Keywords:** Drug discovery, Neuroscience

## Abstract

Orexin, a neuropeptide, performs various physiological functions, including the regulation of emotion, feeding, metabolism, respiration, and sleep/wakefulness, by activating the orexin 1 receptor and orexin 2 receptor (OX2R). Owing to the pivotal role of OX2R in wakefulness and other biological functions, OX2R agonists are being developed. A detailed understanding of OX2R protein distribution is essential for determining the mechanisms of action of OX2R agonists; however, this has been hindered by the lack of selective antibodies. In this study, we first confirmed the OX2R-selective binding of [^3^H]-EMPA in in vitro autoradiography studies, using brain slices from OX2R knockout mice and their wild-type littermates. Subsequently, OX2R protein distribution in rats was comprehensively assessed in 51 brain regions and 10 peripheral tissues using in vitro autoradiography with [^3^H]-EMPA. The widespread distribution of OX2R protein, including that in previously unrecognized regions of the retrosplenial cortex, was identified. In contrast, OX2R protein expression was negligible/very low in peripheral tissues, suggesting that orexin exerts OX2R-dependent physiological functions primarily through activation of the central nervous system. These findings will be useful for understanding the wide range of biological functions of OX2R and the application of OX2R agonists in various disorders.

## Introduction

Orexin, also known as hypocretin, was initially discovered as a regulator of feeding behavior in the hypothalamus^1,2^. Two endogenous orexin peptides, orexin-A and orexin-B, are cleaved from the single precursor prepro-orexin peptide^1,2^. Despite their restricted localization in the lateral hypothalamus, orexin neurons form projections throughout the brain^3,4^. At the terminal, they activate two G protein-coupled receptors: orexin 1 receptor (OX1R) and orexin 2 receptor (OX2R)^1^. OX1R has a greater binding affinity for orexin-A than orexin-B, whereas OX2R has a similar affinity for both peptides^1^. The loss of orexin neurons causes narcolepsy type 1 (NT1), characterized by excessive daytime sleepiness and cataplexy^5–9^. OX2R knockout (KO) mice, but not OX1R KO mice, show NT1-like phenotypes, including clear fragmentation of wakefulness and cataplexy-like episodes^10,11^. In addition, a null mutation in the *OX2R* gene produced a distinct narcolepsy-like phenotype in dogs^12^. Therefore, OX2R appears to play a pivotal role in the pathophysiology of NT1. Apart from regulating wakefulness and cataplexy, orexin is also implicated in multiple biological functions, including emotion, emotional memory, motivation, reward system, feeding, energy homeostasis/metabolism, stress responses, and autonomic function^10,13,14^.

We have recently discovered highly selective OX2R agonists^15^; these are currently under clinical investigation as novel treatment agents for NT1 and other disorders associated with hypersomnia. Considering the broad spectrum of biological functions of orexin, the profiling of OX2R expression in conjunction with its functional analysis could help maximize the therapeutic value of OX2R agonists. Comprehensive *OX2R* mRNA expression analysis has revealed the widespread distribution of OX2R in the central nervous system, with particularly high expression in the hippocampus as well as in various nuclei in the hypothalamus, and undetectable expression in the cerebellum^16,17^. In addition, the expression of *OX2R* mRNA in some peripheral tissues has been reported in a previous study^18^. The expression profiling of OX2R protein has proven to be more beneficial than that of *OX2R* mRNA for the understanding of the pharmacological functions of OX2R agonists. However, research on this topic has been hindered thus far because of the lack of OX2R-selective antibodies.

In vitro autoradiography is a useful method for evaluating the distribution and abundance of receptor proteins when a highly selective radioligand is available^19^. *N*-ethyl-2-[(6-methoxy-pyridin-3-yl)-(toluene-2-sulfonyl)-amino]-*N*-pyridin-3-ylmethyl-acetamide (EMPA) was previously reported to be a high-affinity, reversible, and OX2R-selective antagonist^20^. Nonetheless, its specificity for OX2R has not been fully investigated in in vitro autoradiography studies.

We assessed the selectivity of [^3^H]-EMPA for OX2R protein in in vitro autoradiography experiments using brain sections from OX2R KO mice and their wild-type (WT) littermates. Under similar conditions using [^3^H]-EMPA as an OX2R-selective radioligand, OX2R protein distribution was comprehensively evaluated in 51 brain regions and 10 peripheral tissues of rats, and OX2R protein and mRNA distributions were compared using previously reported mRNA expression data. Our data showed the extensive distribution of OX2R protein expression, including previously unrecognized OX2R expression, in the rat brain, whereas the peripheral tissues had nearly undetectable or very low levels of the protein. Although the elucidation of OX2R function in each region will require further investigation, our results may provide essential information on the therapeutic effects and side effects of OX2R-selective agonists.

## Results

### Selectivity of [^3^H]-EMPA for OX2R in in vitro autoradiography

**Figure 1:**
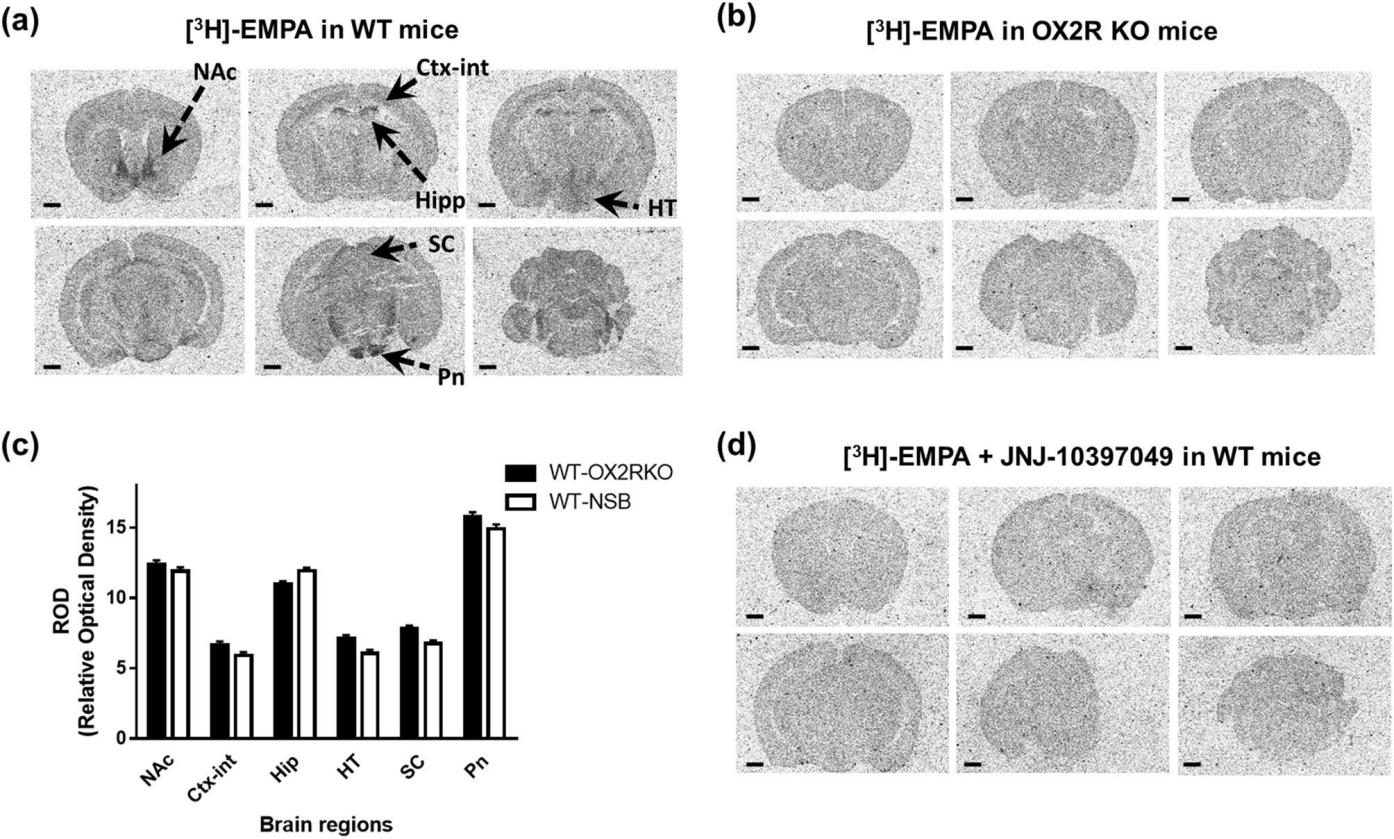
Selective binding of [^3^H]-EMPA to OX2R assessed using brain sections isolated from wild-type (WT) and OX2R knockout (KO) mice. (**a**) Representative autoradiograms evaluating [^3^H]-EMPA binding in six coronal brain sections isolated from WT mice are depicted. All the brain samples were collected at approximately the same time (zeitgeber time (ZT) 6‒8). Scale bar represents 1 mm. (**b**) Representative autoradiograms evaluating [^3^H]-EMPA binding in six coronal brain sections obtained from OX2R KO mice are depicted. All the brain samples were collected at approximately the same time (ZT6‒8). Scale bar represents 1 mm. (**c**) [^3^H]-EMPA binding was quantified (n = 4) in terms of the relative optical density (ROD) in six regions indicated in (**a**) (*NAc* nucleus accumbens, *Ctx-int* internal layer of cortex, *Hip* hippocampus, *HT* hypothalamus, *SC* superior colliculus, and *Pn* pontine nuclei) using coronal brain sections obtained from OX2R KO mice and their WT littermates. The density of specific binding of [^3^H]-EMPA in WT mice was obtained by calculating the difference between the binding observed in WT mice and OX2R KO mice or by calculating the difference between total binding and non-specific binding (NSB) assessed in the presence of 10 µM JNJ-10397049 in WT mice and is shown using filled-in columns (WT-OX2R KO) or blank columns (WT-NSB), respectively. Data are presented as mean ± standard error of the mean. (**d**) Representative autoradiograms evaluating [^3^H]-EMPA binding in the presence of 10 µM JNJ-10397049 as NSB in six coronal brain sections obtained from WT mice are shown. All brain samples were collected at approximately the same time (ZT6‒8). Scale bar represents 1 mm.

The selectivity of the radioligand is critical for evaluating the distribution and abundance of target proteins using in vitro autoradiography^19^. First, the selectivity of [^3^H]-EMPA for OX2R was evaluated by comparing the distribution of [^3^H]-EMPA in brain sections from OX2R KO mice and their WT littermates. Considering the diurnal fluctuation in orexin levels^21–23^, a corresponding variation in OX2R protein expression was anticipated. Therefore, all animals were sacrificed, and their brains were collected at approximately the same time (zeitgeber time (ZT) 6–8). As shown in the representative autoradiographs of brain sections from WT mice (Fig. 1a), a high density of [^3^H]-EMPA binding was observed in specific brain regions, including the hippocampus, nucleus accumbens, and pontine nuclei. [^3^H]-EMPA binding apparently disappeared in brain sections from OX2R KO mice (Fig. 1b). The specific binding of [^3^H]-EMPA to OX2R was evaluated in terms of the relative optical density (ROD) in six brain regions (nucleus accumbens, internal layer of cortex, hippocampus, hypothalamus, superior colliculus, and pontine nuclei) by calculating the difference in [^3^H]-EMPA binding between the brain sections isolated from WT mice and OX2R KO mice; the latter was considered to represent non-specific binding (NSB) (Fig. 1c). A relatively high density of [^3^H]-EMPA binding was observed in the nucleus accumbens, hippocampus, and pontine nuclei. Brain sections with OX2R KO condition are not always available in every species for assessing NSB to measure the specific binding of [^3^H]-EMPA to OX2R. Therefore, NSB was also assessed in the presence of 10 µM JNJ-10397049, a selective and potent OX2R antagonist with more than 500-fold selectivity over OX1R^24,25^. As shown in the representative autoradiographs with brain sections from WT mice (Fig. 1d), [^3^H]-EMPA binding, as observed in Fig. 1a, disappeared in the presence of 10 µM JNJ-10397049. The specific binding of [^3^H]-EMPA to OX2R in the brain sections from WT mice was calculated by subtracting NSB with 10 µM JNJ-10397049 from the total binding (Fig. 1c). The ROD values based on NSB in the presence of 10 µM JNJ-10397049 or in brain sections from OX2R KO mice were considerably similar in all six regions of the brain that were analyzed (Fig. 1c). Therefore, the binding of [^3^H]-EMPA in the brain sections from WT mice was highly selective for OX2R when NSB was assessed using JNJ-10397049.

### Distribution and abundance of OX2R protein in rat brain sections

**Figure 2:**
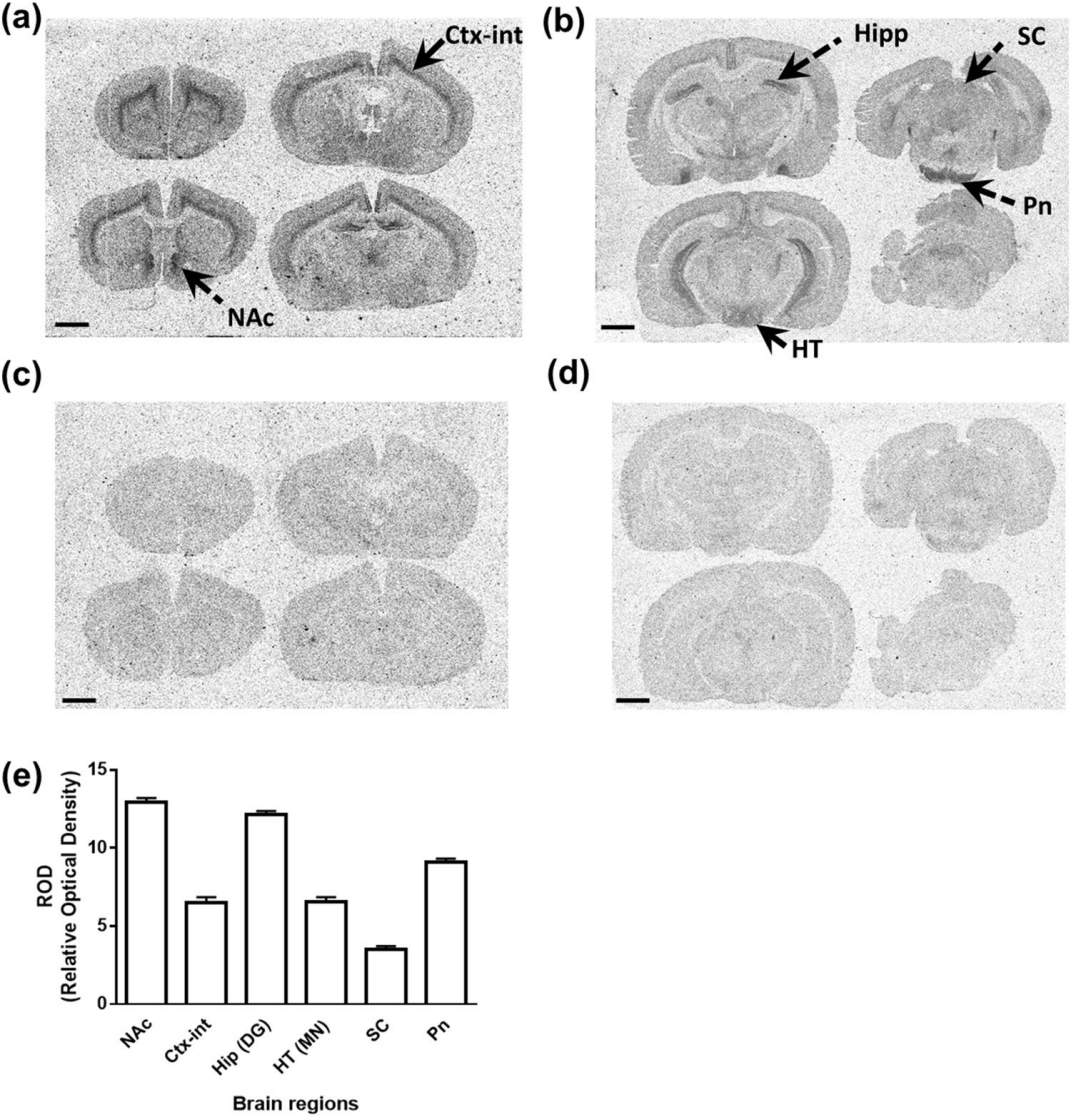
Selective binding of [^3^H]-EMPA to OX2R assessed using rat brain sections. (**a**–**d**) Representative images of OX2R autoradiography in rat brain sections are depicted. All brain samples were collected at approximately the same time (ZT2–3). Non-specific binding (NSB) was analyzed in the presence of 1 µM JNJ-10397049. Total binding (**a**,**b**) and NSB (**c**,**d**) at four frontal brain levels (**a**,**c**) and four caudal brain levels (**b**,**d**) are shown. Scale bar represents 2 mm. (**e**) [^3^H]-EMPA binding was quantified (n = 4) in terms of the relative optical density (ROD) in six regions indicated in (**a**) and (**b**) (*NAc* nucleus accumbens, *Ctx-int* internal layer of cortex, *Hip* (*DG*) hippocampal dentate gyrus, *HT* (*MN*) mammillary nuclei of hypothalamus, *SC* superior colliculus, and *Pn* pontine nuclei) in coronal brain sections. The specific binding of [^3^H]-EMPA is indicated as the difference between total binding and NSB. Data are presented as mean ± standard error of the mean.

To further investigate OX2R protein expression, the distribution and density of [^3^H]-EMPA binding were evaluated in rat brain sections, but not in mouse brain sections, to improve the accuracy of the analysis of [^3^H]-EMPA binding in small brain regions. All brains were collected at approximately the same time (ZT2–3) to avoid potential circadian variations. As shown in Fig. 2a,b, a relatively high density of [^3^H]-EMPA binding was observed in certain regions of the brain, including the nucleus accumbens and hippocampus. This binding almost completely disappeared in the presence of excess JNJ-10397049 (final concentration of 1 µM)^24,25^, as shown in Fig. 2c,d. The density of specific binding of [^3^H]-EMPA to OX2R was evaluated in six regions (nucleus accumbens, internal layer of cortex, hippocampal dentate gyrus, mammillary nuclei of hypothalamus, superior colliculus, and pontine nuclei) of the rat brain sections (Fig. 2e). A relatively high density of [^3^H]-EMPA binding was observed in the nucleus accumbens, hippocampus, and pontine nuclei of the rat brain sections, as seen in the mouse brain sections (Figs. 1c and 2e). Therefore, the experimental conditions were suitable for evaluating OX2R protein distribution and abundance in the rat brain sections using [^3^H]-EMPA.

**Figure 3:**
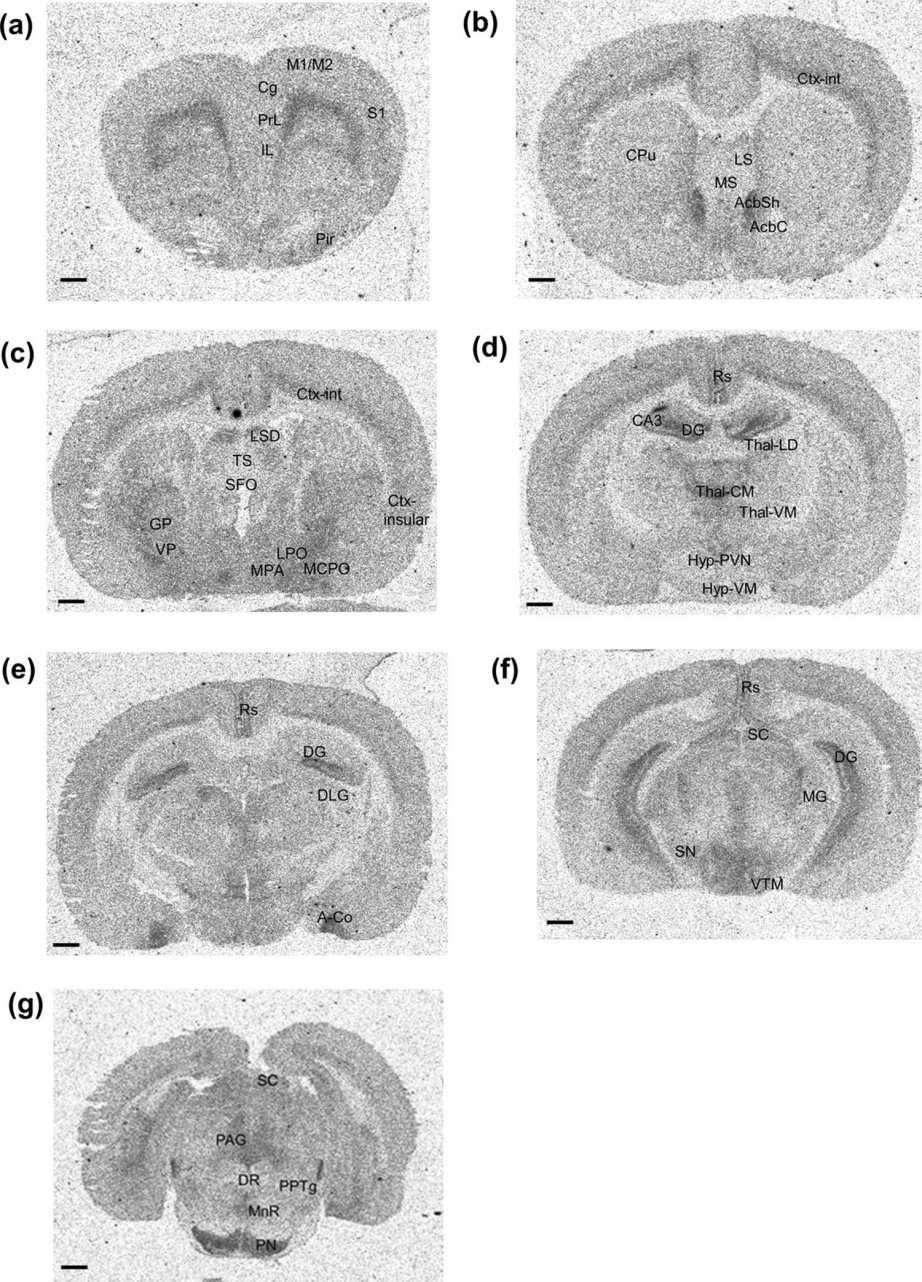
Distribution of [^3^H]-EMPA binding sites in rat brain sections. Representative images of [^3^H]-EMPA total binding in different rat brain sections are shown including: (**a**) infralimbic (IL), prelimbic (PrL), cingulate (Cg), motor (M1/M2), somatosensory (S1), and piriform (Pir) cortical regions; (**b**) internal layer of cortex (Ctx-int), lateral septum (LS), medial septum (MS), caudate putamen (CPu), nucleus accumbens shell (AcbSh), and nucleus accumbens core (AcbC); (**c**) internal layer of cortex (Ctx-int), insular cortex (Ctx- insular), lateral septum dorsal part (LSD), triangular septum (TS), subfornical organ (SFO), globus pallidus (GP), ventral pallidum (VP), and hypothalamus: medial preoptic area (MPA), lateral preoptic area (LPO) and magnocellular preoptic area (MCPO); (**d**) retrosplenial cortex (Rs), hippocampus: dentate gyrus (DG) and CA3, thalamus: laterodorsal nucleus (Thal-LD), central medial nucleus (Thal-CM), ventromedial nucleus (Thal-VM), hypothalamus: ventromedial nucleus (Hyp-VM), and paraventricular nucleus (Hyp-PVN); (**e**) retrosplenial cortex (Rs), hippocampal dentate gyrus (DG), dorsolateral geniculate nucleus of the thalamus (DLG), and cortical amygdala (A-Co). (**f**) Retrosplenial cortex (Rs), hippocampal dentate gyrus (DG), superior colliculus (SC), medial geniculate nucleus of the thalamus (MG), ventral tuberomammillary nucleus of the hypothalamus (VTM), and substantia nigra (SN); (**g**) superior colliculus (SC), periaqueductal gray (PAG), dorsal raphe nucleus (DR), median raphe nucleus (MnR), pedunculopontine tegmental nucleus (PPTg), and pontine nuclei (PN). All brain samples were collected at approximately the same time (ZT2–3). Scale bar represents 1 mm.

Under such conditions, the distribution and density of [^3^H]-EMPA binding were evaluated in 51 regions of the rat brain sections. As expected from the widespread distribution of *OX2R* mRNA expression, an extensive distribution of [^3^H]-EMPA binding was observed in the rat brain sections (Fig. 3, Table 1, and Supplementary Table S1). A very high density of [^3^H]-EMPA binding (ROD ≥ 12) was observed in the hippocampal dentate gyrus, nucleus accumbens (shell), and cortical amygdala. A high density of [^3^H]-EMPA binding (8 ≤ ROD < 12) was also observed in the retrosplenial cortex, ventral pallidum, hypothalamus (paraventricular nucleus and ventral tuberomammillary nucleus), central medial thalamic nucleus, and pontine nuclei. A moderate density of [^3^H]-EMPA binding (4 ≤ ROD < 8) was observed in the piriform cortex, infralimbic cortex, prelimbic cortex, cingulate cortex, internal layer of cortex, hippocampal CA1 and CA3, septum (lateral, medial, and laterodorsal parts), globus pallidus, basolateral amygdala, preoptic area of hypothalamus (magnocellular, lateral, and medial area), mammillary nuclei of hypothalamus, thalamus (medial geniculate nucleus, ventromedial nucleus, and laterodorsal nucleus), periaqueductal gray, dorsal raphe nucleus, median raphe nucleus, locus coeruleus, pedunculopontine tegmental nucleus, laterodorsal tegmental nucleus, Barrington’s nucleus, and pre-Bötzinger complex. A low density of [^3^H]-EMPA binding (1 ≤ ROD < 4) was observed in the motor cortex, somatosensory cortex, insular cortex, cerebellum, triangular septum, caudate putamen, nucleus accumbens (core), subfornical organ, dorsomedial and ventromedial nucleus of the hypothalamus, dorsolateral geniculate nucleus of the thalamus, superior colliculus, and ventral tegmental area. Additionally, negligible [^3^H]-EMPA binding density (ROD < 1) was observed in the substantia nigra. Table 1 also includes the comparison between the qualitative estimates of OX2R protein levels in this study and previously reported *OX2R* mRNA levels^16,17^. Eight brain regions (cingulate cortex, insular cortex, cerebellum, hippocampal CA1 and dentate gyrus, nucleus accumbens shell, cortical amygdala, and ventromedial thalamic nucleus) showed a relatively higher OX2R protein expression than the reported mRNA expression, whereas there were no brain regions with lower OX2R protein expression than the reported mRNA expression^16,17^.

**Table 1:** Relative densities of [^3^H]-EMPA binding for OX2R protein expression in rat brain sections.

Brain area	OX2R protein expression level	Difference from reported OX2R mRNA expression level^16,17^	Brain area	OX2R protein expression level	Difference from reported OX2R mRNA expression level^16,17^
**Cortex**			**Subfornical organ**	+	NR
Piriform cortex	++		**Hypothalamus**		
Infralimbic cortex	++		Dorsomedial nucleus	+	
Retrosplenial cortex	+++	NR	Ventromedial nucleus	+	
Prelimbic cortex	++	NR	Paraventricular nucleus	+++	
Cingulate cortex	++	**↑**	Magnocellular preoptic area	++	
Motor cortex	+	NR	Lateral preoptic area	++	
Somatosensory cortex	+	NR	Medial preoptic area	++	
Internal layer of cortex	++		Ventral tuberomammillary nucleus	+++	
Insular cortex	+	**↑**	Suprachiasmatic nucleus*	+	NR
**Cerebellum**	+	**↑**	Mammillary nuclei	++	
**Hippocampus and septum**			**Thalamus**		
CA1	++	**↑**	Medial geniculate nucleus	++	NR
CA3	++		Dorsolateral geniculate nucleus	+	NR
DG	++++	**↑**	Ventromedial thalamic nucleus	++	**↑**
Lateral septum	++		Central medial thalamic nucleus	+++	
Medial septum	++		Laterodorsal thalamic nucleus	++	NR
Lateral septum dorsal part	++		**Midbrain, pons, and medulla**		
Triangular septum	+	NR	Superior colliculus	+	
**Basal ganglia**			Ventral tegmental area	+	
Caudate putamen	+	NR	Periaqueductal gray	++	
Nucleus accumbens (core)	+		Dorsal raphe nucleus	++	
Nucleus accumbens (shell)	++++	**↑**	Median raphe nucleus	++	
Globus pallidus	++		Pontine nuclei	+++	
Ventral pallidum	+++	NR	Locus coeruleus	++	
Substantia nigra	−		Pedunculopontine tegmental nucleus	++	
**Amygdala**			Laterodorsal tegmental nucleus	++	
Basolateral amygdala	++	NR	Barrington’s nucleus	++	
Cortical amygdala	++++	**↑**	Pre-Bötzinger complex	++	NR

**Figure 4:**
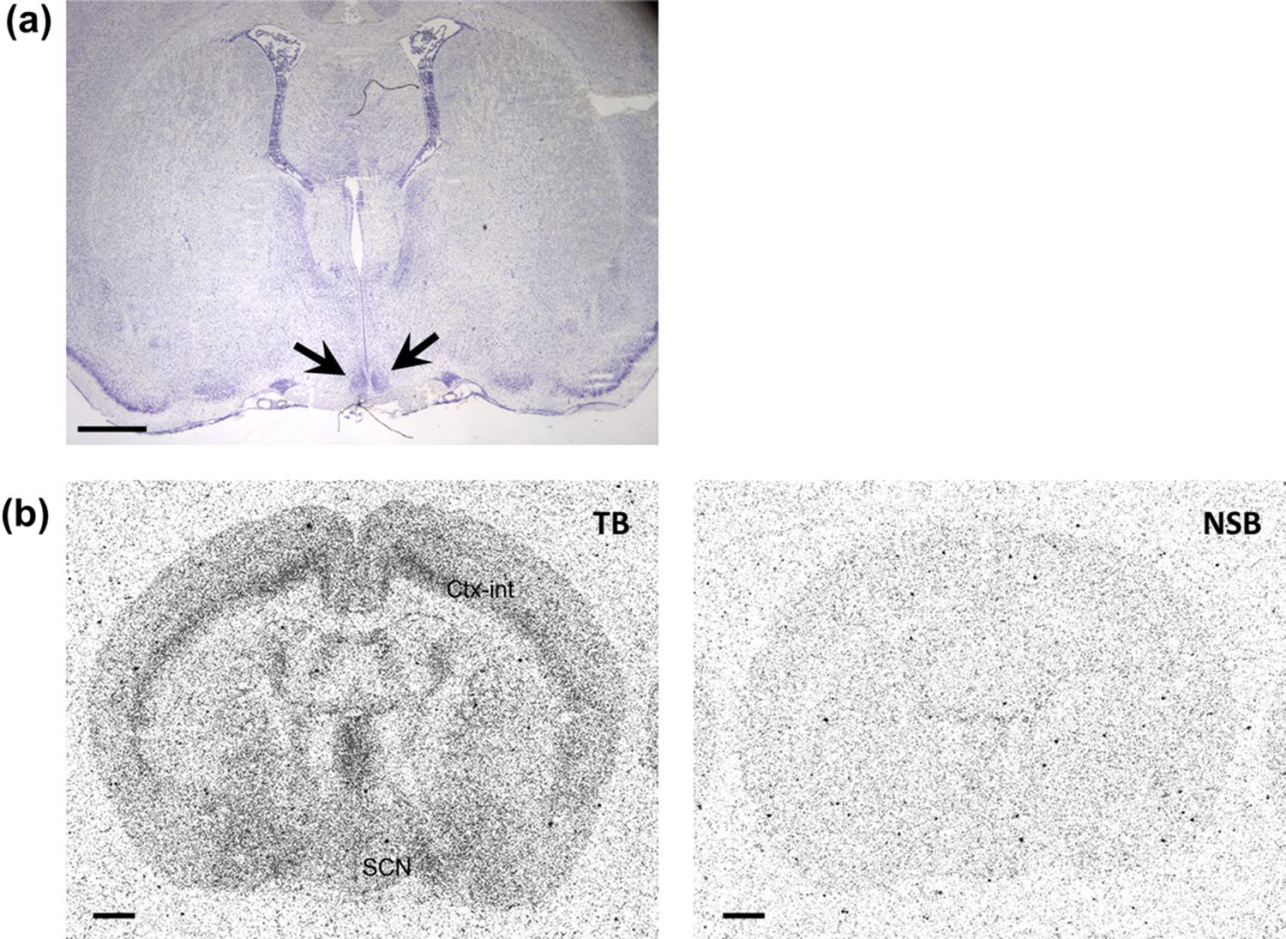
Distribution of [^3^H]-EMPA binding sites in the suprachiasmatic nucleus (SCN) of the hypothalamus in rat brain sections. (**a**) A representative image of Nissl-stained coronal section from a rat brain is shown. Arrow indicates the SCN. (**b**) Representative images of the corresponding autoradiograms evaluating [^3^H]-EMPA binding in the SCN and internal layer of cortex (Ctx-int) are shown. Non-specific binding (NSB) was analyzed in the presence of 1 µM JNJ-10397049. All the rat brain samples were collected at approximately the same time (ZT 2‒3). Scale bar represents 1 mm. *TB* total binding.

Qualitative estimates of OX2R protein expression are presented based on the relative optical density (ROD) values of [^3^H]-EMPA binding (Supplementary Table S1). The following scale was used: ++++, very high density (ROD ≥ 12); +++, high density (8 ≤ ROD < 12); ++, moderate density (4 ≤ ROD < 8); +, low density (1 ≤ ROD < 4); and −, nearly undetectable density (ROD < 1). Difference from the reported *OX2R* mRNA levels^16,17^ is also shown. A relatively higher protein expression than the reported mRNA expression is indicated by “↑”. No reported data for mRNA expression in previous comprehensive assessments is represented as “NR”. *OX2R expression level (ROD value) in the suprachiasmatic nucleus of the hypothalamus was adjusted based on the ROD values in the internal layer of cortex shown in Figs. 3 and 4.

### Distribution and abundance of OX2R protein in the suprachiasmatic nucleus of the hypothalamus in rat brain sections

The suprachiasmatic nucleus (SCN) of the hypothalamus is critical for the circadian regulation. Thus, understanding OX2R expression in the SCN would be important for application of OX2R agonists to circadian rhythm disorders. Unfortunately, brain sections shown in Fig. 3 did not include the SCN; thus, brain sections including the SCN were prepared from a different cohort of rats. All brains were collected at approximately the same time (ZT2–3). NSB was evaluated in the presence of 1 µM JNJ-10397049. Because the SCN is a very small region, Nissl staining with consecutive brain slices were used for its identification (Fig. 4a). Figure 4b shows the corresponding autoradiogram of brain sections including the SCN. To compare the density of [^3^H]-EMPA binding between the SCN shown in Fig. 4b and other brain regions shown in Fig. 3, the ROD value of the internal layer of cortex was used as an internal standard; [^3^H]-EMPA binding in this region was measured in both studies. The adjusted ROD value indicated a low density of [^3^H]-EMPA binding (1 ≤ ROD < 4) in the SCN (Table 1 and Supplementary Table S1).

### Distribution and abundance of OX2R protein in rat peripheral tissue sections

**Figure 5:**
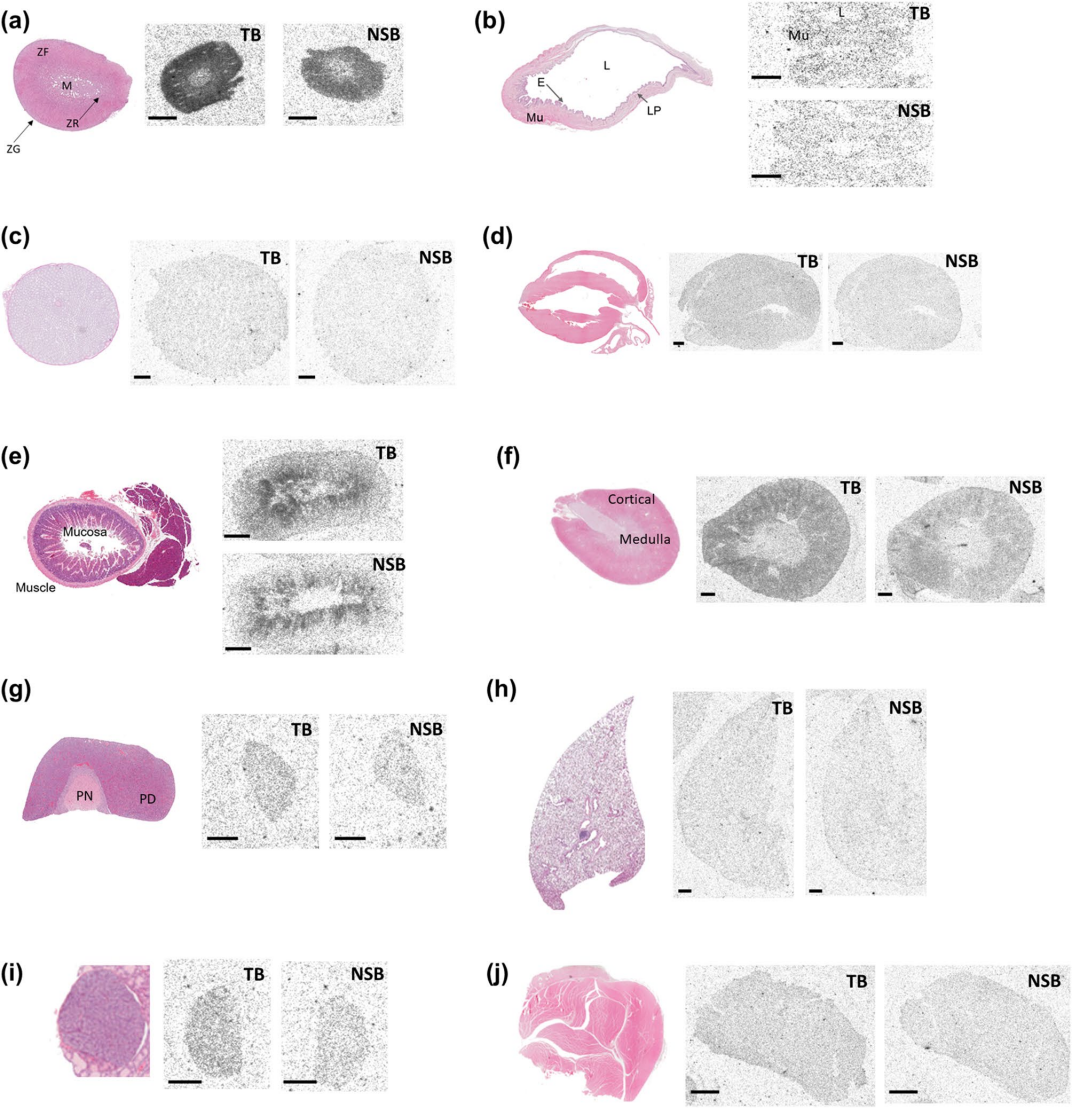
Distribution of [^3^H]-EMPA binding sites in rat peripheral tissue sections. Representative images from hematoxylin–eosin staining and OX2R autoradiography in rat peripheral tissues are shown. All samples were collected at approximately the same time (ZT2–3). Non-specific binding (NSB) was analyzed in the presence of 1 µM JNJ-10397049. Total binding (TB) and NSB in different rat peripheral tissues: adrenal gland (**a**), urinary bladder (**b**), testis (**c**), heart (**d**), gut (**e**), kidney (**f**), pituitary (**g**), lung (**h**), thyroid (**i**), and skeletal muscle (**j**). Scale bar represents 1 mm. *M* medulla, *ZF* zona fasciculate, *ZR* zona reticularis, *ZG* zona glomerulosa, *L* lumen, *Mu* muscle, *E* epithelium, *LP* lamina propria, *PN* pars nervosa, *PD* pars distalis.

The peripheral expression of *OX2R* mRNA, along with OX2R functions, has been reported previously^1,18^. In the present study, we evaluated the distribution and density of [^3^H]-EMPA binding in 10 peripheral tissue sections from rats (Fig. 5, Table 2, and Supplementary Table S2). All tissues or organs were collected at approximately the same time (ZT2–3) to avoid potential circadian variations. NSB was evaluated in the presence of 1 µM JNJ-10397049. A very low density of [^3^H]-EMPA binding (1 ≤ ROD < 2.5) was observed in the adrenal and kidney cortices. A negligible density of [^3^H]-EMPA binding (ROD < 1) was observed in the heart, testis, adrenal medulla, gut (mucosa and muscle), pituitary gland, skeletal muscle, thyroid, lung, and bladder muscle. Table 2 also presents the comparison between the qualitative estimates of OX2R protein levels in this study and the previously reported *OX2R* mRNA levels^1,18^. Compared to the reported OX2R mRNA levels, relatively higher OX2R protein expression was observed in the kidney cortex, whereas relatively lower OX2R protein expression was observed in the adrenal and pituitary glands.

**Table 2:** Relative densities of [^3^H]-EMPA binding for OX2R protein expression in rat peripheral tissue sections.

Peripheral tissue	OX2R protein expression level	Difference from reported OX2R mRNA expression level^1,18^
**Heart**	−	
**Testis**	−	
**Adrenal gland**		↓
Adrenal medulla	−	
Adrenal cortex	+	
**Gut**		
Gut mucosa	−	
Gut muscle	−	
**Kidney cortex**	+	↑
**Pituitary**	−	↓
**Skeletal muscle**	−	
**Thyroid**	−	
**Lung**	−	
**Bladder muscle**	−	NR

Qualitative estimates of OX2R protein expression are presented based on the relative optical density (ROD) values for [^3^H]-EMPA binding (Supplementary Table S2). The following scale was used: +, low density (1 ≤ ROD < 4); −, nearly undetectable density (ROD < 1). Difference from reported OX2R mRNA expression^1,18^ is also depicted. A relatively higher or lower protein expression than the reported mRNA expression is indicated by “↑” or “↓”, respectively. No reported data for mRNA expression in comprehensive assessments is represented as “NR”.

## Discussion

This study presented a comprehensive assessment of OX2R protein distribution and abundance in rat brain sections and peripheral tissue sections using in vitro autoradiography, with [^3^H]-EMPA as an OX2R-selective radioligand. [^3^H]-EMPA binding was abolished in brain slices from OX2R KO mice, suggesting the high selectivity of [^3^H]-EMPA to OX2R. The specific binding densities of [^3^H]-EMPA to OX2R in brain sections from WT mice determined using NSB under excess JNJ-10397049 and in brain sections from OX2R KO mice were quite similar. Therefore, the evaluation of NSB using JNJ-10397049 is useful for estimating OX2R protein levels based on [^3^H]-EMPA binding. Using OX2R-specific autoradiography with [^3^H]-EMPA in rat brain and peripheral tissue sections, we observed widespread OX2R protein distribution in the brain, with high expression in several specific regions, whereas peripheral OX2R protein expression was negligible or very low. The sampling time of tissues from mice (ZT6–8) (Fig. 1) and rats (ZT2–3) (Figs. 2, 3, 4) was different in this study. The purpose of the study using mice was to evaluate the selectivity of [^3^H]-EMPA to OX2R. This objective was achieved by performing all the mice sampling between ZT6–8 and comparing WT mice versus OX2R KO mice or WT mice in the presence and absence of JNJ-10397049. In studies for assessing OX2R protein distribution using rat tissues, all tissue sampling was performed during ZT2–3. Even if OX2R expression is under the circadian variation, the different sampling time between studies using mice and rats should not affect our conclusion.

Consistent with findings from a previously reported autoradiography study^20^, in which [^3^H]-EMPA was used in combination with the OX2R-selective antagonist Cp-5 (exhibiting more than 250-fold selectivity for OX2R over OX1R^26^) for estimating NSB, a high density of [^3^H]-EMPA binding was observed in the nucleus accumbens shell, hippocampus, internal layer of cortex, tuberomammillary nucleus of the hypothalamus, several thalamic nuclei, and pontine nuclei of the rat brain sections, with low density of binding in the nucleus accumbens core. Therefore, autoradiography using [^3^H]-EMPA can be an effective method to accurately and reproducibly measure the OX2R protein distribution.

To our knowledge, two studies have comprehensively assessed *OX2R* mRNA expression in the rat brain^16,17^, and two studies in peripheral tissues in rats^1,18^. Compared with the previously reported *OX2R* mRNA expression levels, we observed higher OX2R protein levels in eight brain regions (cingulate cortex, insular cortex, cerebellum, hippocampal CA1 and dentate gyrus, nucleus accumbens shell, cortical amygdala, and ventromedial thalamic nucleus) and in the kidney cortex, whereas lower levels were observed in two peripheral tissues (adrenal and pituitary glands). The reasons underlying this difference in mRNA and protein levels are unknown. However, besides post-transcriptional regulation, OX2R protein expression at the presynaptic terminals of projecting neurons could contribute to the difference in expression in the target regions of the brain. Nevertheless, the protein distribution profile is more pertinent than the mRNA expression profile for understanding OX2R function. We also observed that OX2R protein expression was relatively high in the retrosplenial cortex and ventral pallidum, and moderate in the prelimbic cortex, basolateral amygdala, medial geniculate, and laterodorsal thalamic nuclei as well as in the pre-Bötzinger complex, with no data on the mRNA expression reported in previous studies^16,17^.

Orexin mediates multiple biological functions, such as arousal, emotion, cognition, reward system, metabolism, and autonomic function^10,13,14^. OX2R expression in the above mentioned brain regions could contribute to the functions of orexin: moderate to high OX2R expression in the retrosplenial cortex, prelimbic cortex, cingulate cortex, insular cortex, hippocampus, basolateral amygdala, cortical amygdala, and some thalamic nuclei related to arousal, emotion, and cognition; nucleus accumbens shell and ventral pallidum related to the motivation/reward system; and the pre-Bötzinger complex correlated with autonomic function. In addition, this expression profiling could further clarify OX2R contribution in additional biological functions. For example, increasing evidence suggests the role of the retrosplenial cortex in conjunction with the hippocampus in memory formation during sleep^27–29^. A relatively high expression of OX2R protein in the retrosplenial cortex and hippocampus might indicate the role of OX2R in cognition not only during wakefulness but also during sleep. The SCN of the hypothalamus, responsible for circadian regulation, exhibited a low level of OX2R protein expression. Orexin neurons receive indirect and direct projections from the SCN^30,31^ to produce diurnal fluctuations in orexin production in rats^32,33^. Reciprocally, orexin-A immunoreactive fibers are distributed in the SCN, wherein orexin-A suppresses the activity of clock cells^34^. If stimulation of OX2R in the SCN can contribute to the circadian regulation, OX2R agonists could be effective in treating disorders associated with circadian rhythm disruption, such as shift-work disorder. Detailed functional studies using OX2R-selective agonists are needed.

Monoaminergic and cholinergic pathways contribute to multiple orexinergic functions, such as arousal and the motivation/reward processes^10^. Orexin neurons densely innervate the following monoaminergic nuclei: noradrenergic locus coeruleus, histaminergic tuberomammillary nucleus of the hypothalamus, serotonergic raphe nuclei, dopaminergic ventral tegmental area, cholinergic pedunculopontine tegmental, and laterodorsal tegmental nuclei. In this study, a relatively high expression of OX2R was observed in both the ventral tuberomammillary nucleus of the hypothalamus and the raphe nuclei (dorsal and median), suggesting OX2R has a significant role in the activity of histaminergic and serotonergic nuclei, which was consistent with previous reports^11,14,35^. mRNA expression in a previous study using in situ hybridization histochemistry showed greater signal ratio in the hypothalamic tuberomammillary nucleus and the raphe nuclei than in the surrounding brain regions^16^, compared with the protein expression in this study. The precise reasons for this difference are unclear, but as discussed above, several reasons could contribute to the difference between mRNA and protein levels. However, strong activation of histaminergic neurons in the hypothalamic tuberomammillary nucleus measured by electrophysiology was observed with an OX2R selective agonist in our previous study^15^, confirming the function of OX2R in this region. Further functional studies using OX2R selective agonists should be considered. In addition, moderate levels of OX2R protein expression were also observed in the locus coeruleus, pedunculopontine tegmental nucleus, and laterodorsal tegmental nucleus. Although OX1R is perceived to play exclusive roles in these regions^11,14,35^, OX2R may also contribute to the activation of noradrenergic and cholinergic neurons. A previous study demonstrated the expression of *OX2R* mRNA in non-monoaminergic neurons in the locus coeruleus and GABAergic neurons in the pedunculopontine tegmental and laterodorsal tegmental nuclei^36^. Therefore, OX2R functions in noradrenergic and cholinergic nuclei should be carefully investigated. The dopaminergic ventral tegmental area showed low levels of OX2R protein expression, suggesting the minor role of OX2R in dopaminergic nuclei.

In rat peripheral tissue sections, OX2R protein expression was mostly negligible, but detected at very low levels in the adrenal cortex, consistent with previously reported mRNA expression^18,37^. Several reports have suggested that orexin stimulates glucocorticoid secretion from adrenocortical cells^38–40^. Glucocorticoid secretion by the adrenal gland is regulated by the hypothalamus–pituitary–adrenal axis, which comprises the paraventricular nucleus of the hypothalamus, the pituitary gland, and the adrenal cortex. As high levels of OX2R protein expression were detected in the paraventricular nucleus of the hypothalamus, OX2R in both the central and peripheral regions may be involved in glucocorticoid secretion. We also observed extremely low OX2R protein expression in the kidney cortex; however, the renal function of OX2R is currently unknown. Detailed functional studies using OX2R-selective agonists, along with an evaluation of the peripheral orexin levels, are needed to further verify OX2R function in these peripheral tissues. The level of blood–brain barrier penetration by orexin is extremely low^41^; thus, the possibility of orexin production in peripheral tissues should also be considered. In fact, low levels of prepro-orexin mRNA were detected in some peripheral tissues, including rat testis^1,18^, and there are some reports on the low levels of plasma orexin-A measured using radioimmunoassay (RIA)^42,43^. However, a recent report indicated that conventional RIA for orexin-A largely measures unauthentic orexin-A-related metabolites^44^. Better methods, including recently reported liquid chromatography mass spectrometry assays^45–47^, should be used for accurately measuring orexin-A levels.

A limitation of this study is that the location of the small brain regions determined using only stereotaxic coordinates may not be precise. In addition, this study did not identify the OX2R-expressing cell types. Further studies are required to identify precise brain regions and cell types with specific markers. In addition, rodents such as mice and rats are nocturnal animals whose sleep/wake structure is polyphasic, whereas primates including human and cynomolgus monkeys are diurnal animals whose sleep/wake structure is monophasic. In future investigations, it would be desirable to perform autoradiography study using primate samples.

In conclusion, our data demonstrated the widespread distribution of OX2R protein expression in the rat brain. In contrast, nearly undetectable or very low levels of OX2R protein expression was observed in the peripheral tissues, indicating that orexin performs OX2R-dependent physiological functions primarily via the activation of the central nervous system. Considering the diverse range of biological functions performed by OX2R, our data would be useful for elucidating the physiological functions and underlying mechanisms of action of OX2R agonists. The evaluation of OX2R protein expression in animal models and human postmortem tissues using in vitro autoradiography with [^3^H]-EMPA could also improve our understanding of the implication of OX2R in various disorders. The clinical development of our OX2R agonists is currently underway (ClinicalTrials.gov Identifier: NCT04091438, NCT04820842, NCT04096560).

## Methods

### Animals

OX2R KO mice and their littermate WT mice were generated and bred in our laboratory^15^. Nineteen-week-old male OX2R KO mice and their littermate WT mice were used to evaluate the selectivity of [^3^H]-EMPA for OX2R. All mice were housed under ethical laboratory conditions (12 h light/dark cycles: lights on at 7:00) with food (CE-2, CLEA Japan Inc.) and water available ad libitum. Animals were maintained and sacrificed according to the guidelines of the Institutional Animal Care and Use Committee (IACUC) of Takeda Pharmaceutical Company Limited, which is accredited by the Association for Assessment and Accreditation of Laboratory Animal Care International (AAALAC).

Male Sprague–Dawley rats (approximately 250 g, Charles River, Italy) were used to evaluate OX2R expression in the brain sections and peripheral tissue sections. All rats were housed under ethical laboratory conditions (12 h light/dark cycles, lights on at 6:00) with food (rat and mouse maintenance diet Altromin R, A. Rieper SpA, Bolzano, Italy) and water available ad libitum.

OX2R expression studies were conducted in Aptuit (Verona) Srl under the approval of the internal Aptuit Committee on Animal Research and Ethics, with authorization issued by the Italian Ministry of Health (Italian Ministry of Health Authorization Project). General procedures for animal care and housing were in accordance with the current AAALAC recommendations.

All animal experiments were performed in accordance with ARRIVE guidelines.

### Chemicals and radiolabeled ligand

[^3^H]-EMPA (specific activity: 79 Ci/mmol for Figs. 1, 2, 3 and 5, 86 Ci/mmol for Fig. 4) was purchased from Novandi Chemistry AB (Södertälje, Sweden). Additionally, JNJ-10397049 was synthesized by Aptuit (Verona) Srl (Verona, Italy), and 2-methylbutane was purchased from Fujifilm Wako Pure Chemical Co. (Osaka, Japan). Entellan was purchased from Merck KGaA (Darmstadt, Germany).

### Preparation of mice brain sections

Immediately following euthanasia, the brains of adult OX2R KO mice and their littermate WT mice (n = 4 per group) were rapidly dissected and promptly frozen in pre-cooled (− 30 to − 20 °C) 2-methylbutane in dry ice. Coronal sections (14 µm-thick) were dissected at six brain levels corresponding to the regions of interest with reference to stereotaxic coordinates^48^ and were mounted on glass slides. The sections were stored at − 80 °C until autoradiography was performed. All animals were sacrificed according to the guidelines of the IACUC of Takeda Pharmaceutical Company Limited, and the brains were collected at approximately the same time (ZT6–8) to avoid any possible diurnal variations.

### Preparation of rat brain and peripheral tissue/organ sections

Immediately following euthanasia, the brains and 10 peripheral tissues or organs of adult male Sprague–Dawley rats (n = 4 per group) were rapidly dissected and promptly frozen in pre-cooled (− 30 °C) 2-methylbutane in dry ice. Brains were dissected into 14 µm-thick coronal sections at different levels corresponding to the regions of interest with reference to stereotaxic coordinates^49^. Subsequently, these sections were mounted on glass slides and stored at − 80 °C until autoradiography was performed. Nissl staining with cresyl violet was performed as described previously^49^ and briefly mentioned as below. Sections were immersed for 5 min in each of the following: xylene (× 2), 100% alcohol (× 2), 95% alcohol, and 75% alcohol. They were subsequently dipped in distilled water and stained in 0.5% cresyl violet for 15–30 min. After being differentiated in water for 3–5 min, they were dehydrated through 70% alcohol, 95% alcohol, and 100% alcohol (2 ×) and coverslipped with Entellan. Furthermore, 10 peripheral tissues or organs were dissected into 14 µm-thick coronal sections and mounted on glass slides. These sections were stored at − 80 °C until autoradiography was performed. During necropsy, parts of the same tissues or organs from all rats were preserved in 10% neutral buffered formalin. After fixation, the tissues were further processed for paraffin embedding into wax blocks, sectioned at a nominal thickness of 35 µm, and stained with hematoxylin–eosin for microscopic examination. All animals were sacrificed, and all samples were collected at approximately the same time (ZT2–3) to avoid any possible diurnal variations.

### OX2R autoradiography

Tissue sections were pre-incubated for 15 min in an assay buffer (25 mM HEPES, 1 mM CaCl_2_, and 5 mM MgCl_2_; pH 7.4) and subsequently incubated for 60 min at room temperature in the assay buffer supplemented with 3 nM [^3^H]-EMPA. Thereafter, the sections were washed twice in the assay buffer at 4 °C for 1 min and rapidly rinsed in Milli-Q water. The sections were then air-dried and exposed to BAS-TR2025 Fuji imaging plates (Fuji Film, Tokyo, Japan) for 9 days. Subsequently, NSB was estimated in terms of the extent of binding in the presence of excess JNJ-10397049 (an OX2R-selective antagonist)^24,25^ (final concentration of 1 µM for the rat study and 10 µM for the mice study). Autoradiograms were generated using a Phospho-Imager Bio-image Analyzer BAS5000 (Fuji Photo Film Co., Japan). The radioactivity of the region of interest was analyzed as photostimulated luminescence per mm^2^ using computer-assisted microdensitometry (MCID basic, Imaging Research, Canada) and was expressed in terms of ROD values.

Four slides were used for each brain or peripheral tissue: three slides for the determination of total binding and one slide for the evaluation of NSB. For each region of interest, the signal density of total binding was measured in duplicate in each section and averaged from the values for three consecutive sections on different slides.

Specific binding was calculated by subtracting NSB from total binding. Because of the slight differences in the background binding level on the glass slides (outside the sections), the following analysis was performed to calculate the specific binding to correct any differences in experimental conditions among the slides: specific binding = (total binding − background) − (NSB − background). The background binding level on each glass slide was calculated as the average of nine spot areas outside the sections on the glass slide.

Specific binding in the SCN was adjusted based on the ROD value in the internal layer of the cortex as an internal control. The mean ROD value in the internal layer of cortex was 6.52 in the study indicated in Fig. 3 and 10.02 in the study shown in Fig. 4. Therefore, ROD values in the SCN in the study shown in Fig. 4 were adjusted by multiplying by 6.52/10.02. Then, the mean and standard error of the mean in the SCN were calculated.

### Comparison of protein expression with reported mRNA expression

Protein expression observed in this study and previously reported mRNA expression^1,16–18^ were compared. Protein expression was defined to be higher than mRNA expression if the former was more than low (+) in regions with previously reported undetectable mRNA expression or if it was clearly higher than previously reported mRNA expression (+ for mRNA vs. +++/++++ for protein or ++ for mRNA vs. ++++ for protein). Conversely, lower protein expression than mRNA expression was determined based on undetectable protein expression in regions where mRNA expression was observed in previous studies. In addition, previous studies on peripheral *OX2R* mRNA expression did not include any qualitative assessments. The OX2R protein expression level in the adrenal cortex was very low in our study, whereas the *OX2R* mRNA expression level in the adrenal cortex was the highest among the expression levels in the whole brain and 14 peripheral tissues in a previous study^18^. Therefore, our study reported a decrease in protein expression compared to mRNA expression in the adrenal cortex.

### Ethics declarations

The care and use of the animals and the experimental protocols used in this study were approved by the Institutional Animal Care and Use Committee of Takeda Pharmaceutical Company Limited or by the internal Aptuit Committee on Animal Research and Ethics with authorization issued by the Italian Ministry of Health (Italian Ministry of Health Authorization Project). General procedures for animal care and housing were in accordance with the current AAALAC recommendations. All animal experiments were performed in accordance with ARRIVE guidelines.

## Supplementary Information

Supplementary Tables.

## Data Availability

All raw data and materials are available on request.
