# Supplementary material for: Orexin 2 receptor (OX2R) protein distribution measured by autoradiography using radiolabeled OX2R-selective antagonist EMPA in rodent brain and peripheral tissues
Source: Sci Rep. 2022 May 19;12:8473. doi: 10.1038/s41598-022-12601-x (PMC9120030; doi:10.1038/s41598-022-12601-x)
Supplement: Supplementary file 1 — Supplementary Tables. [file 41598_2022_12601_MOESM1_ESM.pdf]

Supplementary Information (Supplementary tables) for

Orexin 2 receptor (OX2R) protein distribution measured by autoradiography using radiolabeled OX2R-selective antagonist EMPA in rodent brain and peripheral tissues

Kayo Mitsukawa & Haruhide Kimura*

Affiliations:

Research, Takeda Pharmaceutical Company Limited, 26-1, Muraoka-Higashi 2-Chome, Fujisawa, Kanagawa 251-8555, Japan

*Correspondence: haruhide.kimura@takeda.com

Supplementary Table S1. Values of relative optical density in various brain regions

* The value in the suprachiasmatic nucleus was adjusted based on the values in the internal layer of cortex in studies indicated in Fig. 3 and Fig. 4.

Brain Area		Relative optical density	
		Mean	Standard error of the mean
Cortex	Piriform cortex	6.10	0.42
	Infralimbic cortex	4.07	0.31
	Retrosplenial cortex	10.50	0.55
	Prelimbic cortex	4.08	0.24
	Cingulate cortex	5.78	0.29
	Motor cortex	3.93	0.25
	Somatosensory cortex	3.12	0.32
	Internal layer of cortex	6.52	0.32
	Insular cortex	3.90	0.27
Cerebellum		1.52	0.40
Hippocampus and Septum	CA1	4.06	0.34
	CA3	6.44	0.53
	DG	12.15	0.22
	Lateral septum	4.49	0.22
	Medial septum	4.61	0.33
	Lateral septum dorsal part	6.20	0.35
	Triangular septum	2.04	0.27
Basal ganglia	Caudate putamen	3.04	0.30
	Nucleus accumbens (core)	3.86	0.18
	Nucleus accumbens (shell)	12.94	0.25
	Globus pallidus	6.68	0.41
	Ventral pallidum	9.49	0.27
	Substantia nigra	0.59	0.20
Amygdala	Basolateral amygdala	5.21	0.27
	Cortical amygdala	12.43	0.59
Subfornical organ		2.67	0.30
Hypothalamus	Dorsomedial nucleus	1.87	0.46
	Ventromedial nucleus	1.08	0.21
	Paraventricular nucleus	8.03	0.20
	Magnocellular preoptic area	7.50	0.61
	Lateral preoptic area	6.70	0.60
	Medial preoptic area	4.10	0.41
	Ventral tuberomammillary nucleus	8.20	0.57
	Suprachiasmatic nucleus*	2.52	0.25
	Mammillary nuclei	6.53	0.31
Thalamus	Medial geniculate nucleus	5.45	0.35
	Dorsolateral geniculate nucleus	3.05	0.60
	Ventromedial nucleus	5.17	0.77
	Central medial nucleus	10.72	0.31
	Laterodorsal nucleus	7.28	0.30

Midbrain, Pons and Medulla	Superior colliculus	3.48	0.24
	Ventral tegmental area	2.61	0.30
	Periaqueductal gray	4.69	0.44
	Dorsal raphe nucleus	6.10	0.38
	Median raphe nucleus	5.32	0.61
	Pontine nuclei	9.07	0.25
	Locus coeruleus	4.77	0.41
	Pedunculopontine tegmental nucleus	4.02	0.46
	Laterodorsal tegmental nucleus	4.40	0.41
	Barrington's nucleus	4.13	0.44
	Pre-Bötzinger complex	6.84	0.58

Supplementary Table S2. Values of relative optical density in peripheral tissues

Peripheral tissues		Relative optical density	
		Mean	Standard error of the mean
Heart		0.77	0.16
Testis		-0.06	0.16
Adrenal gland	Medulla	0.23	0.21
	Cortex	2.27	0.49
Gut	Mucosa	0.13	0.31
	Muscle	0.33	0.11
Kidney	Cortex	1.46	0.32
Pituitary		0.60	0.14
Skeletal muscle		0.07	0.14
Thyroid		0.30	0.17
Lung		0.02	0.13
Bladder	Muscle	0.24	0.15
